# RNA interference for CFTR attenuates lung fluid absorption at birth in rats

**DOI:** 10.1186/1465-9921-9-55

**Published:** 2008-07-24

**Authors:** Tianbo Li, Shyny Koshy, Hans G Folkesson

**Affiliations:** 1 Department of Integrative Medical Sciences, Northeastern Ohio Universities Colleges of Medicine and Pharmacy, Rootstown, OH 44272-0095, USA

## Abstract

**Background:**

Small interfering RNA (siRNA) against αENaC (α-subunit of the epithelial Na channel) and CFTR (cystic fibrosis transmembrane conductance regulator) was used to explore ENaC and CTFR function in newborn rat lungs.

**Methods:**

Twenty-four hours after trans-thoracic intrapulmonary (ttip) injection of siRNA-generating plasmid DNA (pSi-0, pSi-4, or pSi-C_2_), we measured CFTR and ENaC expression, extravascular lung water, and mortality.

**Results:**

αENaC and CFTR mRNA and protein decreased by ~80% and ~85%, respectively, following αENaC and CFTR silencing. Extravascular lung water and mortality increased after αENaC and CFTR-silencing. In pSi-C_2_-transfected isolated DLE cells there were attenuated CFTR mRNA and protein. In pSi-4-transfected DLE cells αENaC mRNA and protein were both reduced. Interestingly, CFTR-silencing also reduced αENaC mRNA and protein. αENaC silencing, on the other hand, only slightly reduced CFTR mRNA and protein.

**Conclusion:**

Thus, ENaC and CFTR are both involved in the fluid secretion to absorption conversion around at birth.

## Background

Fetal lungs are filled with fluid that is produced and secreted by the pulmonary epithelium and linked to Na-coupled Cl secretion *in utero*. This fluid must be rapidly removed at birth for adequate gas exchange across the alveolar epithelium-endothelium at birth to occur. Failure to clear fetal lung fluid has been linked to preterm birth, inherited genetic diseases, and inflammation and may increase the risk of hypoxic injury to vital organs in newborn infants; such injuries representing ~4% of infant fatalities in 2002 [1].

Lung fluid absorption and secretion have been intensively studied [2-7]. Apical amiloride-sensitive epithelial Na channels (ENaC) [8,9] and basolateral Na,K-ATPases [10,11] have been demonstrated as key proteins for vectorial Na transport and lung fluid absorption. Recent studies in fetal rats found an accelerated lung fluid absorption between birth and 40 h postnatal age [12]. Lung Cl transport is traditionally associated with fluid secretion during lung development [2]. Studies of cultured alveolar type II epithelial cells suggest that cystic fibrosis transmembrane conductance regulator-(CFTR)-mediated Cl transport may also be involved [13,14]. There are, however, still some questions to CFTR involvement since these studies rely on cultured cells of uncertain phenotype and did not address the possibility that fluid transport may involve multiple different epithelial cell types, including alveolar epithelial type I cells [15,16] and distal airway epithelial cells [17,18]. In fact, the Na transport machinery involved in lung fluid absorption as well as CFTR are present in both alveolar epithelial type I and II cells [19].

ENaC and CFTR functions have been assessed in αENaC^-/- ^[8,20] and CFTR^-/- ^[21] mice, but these results may be confounded by various *in vivo *compensatory mechanisms. In the present study, our first aim was to adapt and use our recently developed RNA interference (RNAi) technique [9] to silence CFTR in newborn rats by trans-thoracic intrapulmonary (ttip) injection and to explore functional *in vivo *responses to the CFTR-silencing. Thus, we determined extravascular lung water and newborn mortality after CFTR-silencing. Our second aim was to use RNAi technology to silence CFTR in primary distal lung epithelial cells (DLE cells) and to compare the effects with the *in vivo *situation. Our third aim was to determine if CFTR-silencing affected lung αENaC expression and if αENaC silencing affected lung CFTR expression *in vivo *and *in vitro*.

## Materials and methods

### Animals

Timed-pregnant Sprague-Dawley rats (wt 200–250 g, *N *= 28; Charles River, Wilmington, MA) were used in the study. The rats were housed separately in their cages in a temperature- and humidity-controlled environment (20 ± 2°C and 55 ± 10% relative humidity). The rats were kept at a 12:12 h day-night rhythm and had free access to standard rat chow (Purina, Copley Feed, Copley, OH) and tap water. All studies were reviewed and approved by the Institutional Animal Care and Use Committee (IACUC) at the Northeastern Ohio Universities Colleges of Medicine and Pharmacy, Rootstown, OH.

### Plasmid construction

siRNA-generating plasmids were constructed using a commercial plasmid (pSilencer 3.0-H1; Ambion, Austin, TX) with standard techniques [22]. Selected recombinants were sequenced (CEQ 2000XL; Beckman, Palo Alto, CA) to verify correct oligonucleotide frames and sequences. Plasmid DNA (pDNA) was amplified in *Escherichia coli *DH5α and purified using the Wizard^® ^PureFection pDNA Purification System (Promega Co., Madison, WI). This pDNA isolation kit has a specific resin-binding procedure to remove endotoxin from the pDNA. After isolation and purification, pDNA concentration and purity (ratio 1.7–1.8) was measured at 260/280 nm and samples were stored at -80°C.

#### CFTR

Rat CFTR mRNA [GenBank:XM_347229] secondary folding structure was predicted based on the principle of minimizing free energy, using RNA structure v. 3.71 software [9]. Two 19-nucleotide regions from cDNA, 3872–3892 bp and 3458–3478 bp, were selected and designed as targets for rat CFTR specific siRNA-generating pDNA, named pSi-C_1 _and pSi-C_2_, respectively. Each target sequence was specific and did not match other sequences in the GenBank. For construction of siRNA-generating pDNA, two complementary oligonucleotides (forward and reverse), containing a sense strand, followed by a short spacer (5'-TTCAAGAGA-3'), an antisense strand, and a RNA polymerase III termination signal (5'-TTTTTTGGAAA-3'), were synthesized, annealed, and ligated into pSilencer 3.0-H1. Synthesized oligonucleotides with *Bam*HI and *Hind*III overhangs were **pSi-C**_1_, forward 5'-GATCCGTGGAGAGATGAAGAAATATTTCAAGAGAATATTTCTTCATCTCTCCATTTTTTGGAAA-3' **pSi-C**_1_, reverse 5'-GCTTTTCCAAAAAATGGAGAGATGAAGAAATATTCTCTTGAAATATTTCTTCATCTCTCCACG-3'; **pSi-C**_2_, forward 5'-ATCCGAAAGTATATGTACCAAGATTCAAGAGATCTTGGTACATATACTTTCTTTTTTGGAAA-3', **pSi-C**_2_, reverse 5'-AGCTTTTCCAAAAAAGAAAGTATATGTACCAAGATCTCTTGAATCTTGGTACATATACTTTCG-3'. As negative control we used a non-silencing sequence, 5'-GATCCGTTACACTTTTTTGGAAA-3' (scramble, which does not correspond to any known transcript) with *Bam*H1 and *Hind*III overhangs, also inserted in pSilencer 3.0-H1, named pSi-0.

#### αENaC

Rat αENaC mRNA [GenBank:NM_031548] secondary folding structure was also predicted based on the principle of minimizing free energy as above. In our earlier study [9], we generated four pDNA constructs named pSi-1 – pSi-4 for αENaC silencing. Pilot studies [9] demonstrated that pSi-4 was the most effective pDNA construct and was selected for these studies. The pSi-4 sequence corresponds to rat αENaC cDNA nucleotide positions 1617–1635, is specific for rat αENaC and do not match other GenBank sequences. For construction of siRNA-generating pDNA, the same procedure as above was followed. The two oligonucleotides were: **pSi-4**, forward: 5'-GATCCGTTACACTATTAACAACAAATTCAAGAGATTTGTTGTTAATAGTGTAATTT TTTGGAAA-3', **pSi-4**, reverse: 5-AGCTTTTCCAAAAAATTACACTATTAACAACAAATCTCTTGAATTTGTTGTTAATAG TGTAACG-3'. We also used the same negative control, pSi-0.

### Solutions

After measuring DNA concentration, the pDNA solution was either concentrated by a vacuum centrifuge (SVC100H; Savant Instrument Inc., Farmingdale, NY) or diluted with sterile deionized water to the required concentration. pDNA solution osmolality was measured by a Vapor Pressure Osmometer 5500 (Wescor Inc., Logan, UT), and if needed adjusted with sterile NaCl or deionized water to 100 mOsm. The pDNA solution was freshly prepared by mixing pDNA containing either pSi-C_1_, pSi-C_2_, pSi-4, or pSi-0 with Lipofectamine 2000™ (Invitrogen, Carlsbad, CA), under optimized transfection conditions [9]: pDNA (μg): Lipofectamine 2000™ (μl) ratio 1:1, generating a pDNA solution with the concentration 4 μg/g body wt in a final volume of 40 μl/g body wt for each newborn rat.

### pDNA delivery

Timed-pregnant rats were observed for signs of labor and delivery. Newborn rats were removed from the dams within 1 h after birth. Freshly prepared pDNA/Lipofectamine solution was delivered trans-thoracically via the left pleural cavity to the lungs using a 30-G needle in a volume of 40 μl/g body wt [23]. Newborn rats were placed on a 37°C temperature-controlled pad after pDNA injection. The newborn rats were then allowed to recover in cages with their respective dams where they remained for the 24-h study.

### Specific protocols

All newborn rats were pretreated with scramble pDNA (pSi-0), specific αENaC-silencing pDNA (pSi-4), or specific CFTR-silencing pDNA solution (pSi-C_1 _or pSi-C_2_) for 24 h as described above and divided into the following groups. Mortality was recorded in all experimental groups. *Untreated: *Newborn rat lungs were excised for either extravascular lung water or RT-PCR and western blot studies (*N *= 18). *Pilot studies: *For CFTR we tested the two candidate sequences in newborn rats, pSi-C_1 _(*N *= 4) and pSi-C_2 _(*N *= 4), and based on the efficiency data from our pilot studies, we selected pSi-C_2 _as siRNA-generating pDNA for CFTR. *Control: *Newborn rats were ttip injected with irrelevant pDNA (pSi-0, *N *= 32). Lungs were excised for either extravascular lung water or RT-PCR and western blot studies. *CFTR siRNA: *Newborn rats were ttip injected with CFTR siRNA-generating pDNA (pSi-C_2_, *N *= 63). Lungs were excised for either extravascular lung water or RT-PCR and western blot studies. α*ENaC siRNA: *Newborn rats were ttip injected with αENaC siRNA-generating pDNA (pSi-4, *N *= 40). Lungs were excised for either extravascular lung water or RT-PCR and western blot studies.

### DLE cell isolation, culture, and RNAi localization

DLE cells were isolated from GD21 (GD = gestation day; term = 22 days; *N *= 75 fetuses from 6 dams) rat fetuses [24]. Briefly, dams were anesthetized with heparinized (1,000 U) pentobarbital sodium (50 mg/kg body wt) intraperitoneally and placed in temperature-controlled environments. Rat fetuses were delivered one-by-one via abdominal hysterotomy. Between deliveries the uterus was kept closed by a non-injurious hemostat. Fetal lungs and hearts were excised *en bloc *(heart was removed and discarded) immediately after fetal decapitation. Lungs from each litter were pooled, rinsed twice in ice-cold HBSS (w/o Mg & Ca), and minced to <1 mm^3^. Fetal lung tissue was digested in HBSS containing 0.125% trypsin (Mediatech, Herndon, VA) and 25 μg/ml DNase I (MP Biochemicals, Aurora, OH) 20 min at 37°C. After 20 min, collagenase (USB Co., Cleveland, OH) and additional DNase I were added to final concentrations of 0.1% and 50 μg/ml, respectively, and digestion was continued for 20 min. Enzymes were neutralized by adding 2 ml FBS (fetal bovine serum; Atlanta Biologicals, Lawrenceville, GA) at 4°C. Cell suspensions were transferred to new tubes by tituration to break up cell clumps. Dispersed cell solutions were filtered through 100 μm cell strainers (Becton Dickinson Labware, Franklin Lakes, NJ), and then through 70 μm cell strainers. DLE cells were collected by centrifugation (420 *g*; 6 min) and resuspended in 15 ml DMEM/F-12 (Dulbecco's modified Eagle medium/F-12 50/50; Cellgro, Herndon, VA). The DLE cells were purified by differential adherence steps. Cells were plated 2 × 30 min to remove fibroblasts. Cell yield was determined by a Beckman Coulter Z1 Coulter particle counter. The purity of the isolated DLE cells was on average 85–90% DLE cells. Isolated DLE cells were seeded on 6-well plates (Corning, Acton, MA) at 10^5 ^cells/cm^2 ^densities. All cells were submersion cultured in DMEM/F-12 with 10% FBS in an atmosphere of 5% CO_2_, 21% O_2_, 74% N_2 _with 95% humidity.

DLE cells were also isolated from newborn rats ttip injected with pSi-0 or pSi-C_2 _(each *N *= 6) for 24 h. These DLE cells were isolated following the same technique [24] with some modifications. Lungs and hearts were excised *en bloc *(heart was removed and discarded) immediately after fetal decapitation. Blood was collected. Lungs from each litter were pooled, rinsed twice in ice-cold HBSS (w/o Mg & Ca), and minced to ~1 mm^3^. Lung tissue was then processed as described above. For RNAi localization, cells were collected by centrifugation 5 min at 1000 *g *and snap-frozen in liquid nitrogen for down-stream analyses.

### DLE cell transfection

Isolated DLE cells were transfected at 60%–80% confluency (1 day after plating) in 6-well plates using the prepared pDNA/Lipofectamine solution according to the manufacturer. Each transfection solution contained 10 μg of pDNA, pSi-C_2_, pSi-4, or pSi-0, and 10 μl Lipofectamine 2000™ in a total volume of 2 ml. After 6 h incubation, the transfection solution was replaced with 10% FBS containing DMEM/F-12 medium. Twenty-four hours later, the cells were lysed by appropriate lysis buffer depending on down-stream analyses.

### PCR

A polymerase chain reaction (PCR) was used to detect pDNA after *in vivo *and *in vitro *pSi-0 transfection. Template DNA was isolated from lung tissue, DLE cells, and for control also kidney tissue using a DNA isolation kit (Promega Co., Madison, WI). A primer pair was synthesized targeting a pSi-0- specific sequence, **pSi-0+**: 5'-CACTCGGATCCGTTACACTT-3', and **pSi-0-**: 5'-TAGTCCTGTCGGGTTTCG-3'. PCR was done with a PCR Master Mix kit (Promega). Reaction volume was 25 μl, with 50 ng of template DNA and 0.1 μM of each primer added, under optimized conditions: 95°C 30 sec, 55°C for 30 sec, 72°C for 1.5 min, for 30 cycles, and final extension for 5 min at 72°C. PCR amplification would yield a 127-bp pSi-0-specific fragment. PCR products were resolved in 1.5% agarose gel containing 1 μg/ml ethidium bromide. Gels were scanned by a Typhoon 8610 scanner (Molecular Dynamics).

### RT-PCR

Total RNA was extracted from lung tissue, isolated DLE cells, and kidney tissue using a Versagene RNA isolation kit from Gentra (Minneapolis, MN). RNA yield and purity was determined spectrophotometrically at 260/280 nm and RNA integrity was verified by agarose gel electrophoresis. A competitive reverse transcriptase polymerase chain reaction (RT-PCR) was carried out using the One-Step RT-PCR kit (EMD, San Diego, CA). Total reaction volume was 25 μl, containing 50 ng total RNA, 1 × PCR buffer, 0.2 mM of each dNTP, 2.5 mM MgSO_4_, 0.1 μM of each primer and 1.5 U rTth DNA polymerase. The RT-PCR was optimized: 60°C 30 min reverse transcription, followed by 40 cycles at 94°C 45 sec, 60°C 2 min, and final extension 7 min at 60°C. We tested in preliminary experiments 30 and 40 amplification cycles for pSi-4 [9]. We elected to use 40 cycles after analysis of outcome versus number of cycles and because we found that this amplification generated repeatable results. Three primer pairs (+, sense; -, antisense) were derived from GenBank sequences, and synthesized for competitive RT-PCR: α**ENaC **[GenBank:NM_031548], ENa+: 5'-CATGATGTACTGGCAGTTCGC-3' (731–751), ENa-: 5'-TCCCTTGGGCTTAGGGTAGAAG-3' (1751–1772); **CFTR **[GenBank:XM_347229], CF+: 5'-ACTTACTTTGAAACCCTATTCC-3' (3157–3178), CF-: 5'-AAGGCTTGTCTTAGAACTCG-3' (4102–4121); **GAPDH **[GenBank:NM_017008], GAPD+: 5'-ACCACAGTCCATGCCATCAC-3' (1369–1388), GAPD-: 5'-TCCACCACCCTGTTGCTGTA-3' (1801–1820). Amplification of this competitive RT-PCR yields a 1042-bp αENaC fragment, a 965-bp CFTR fragment, and a 452-bp GAPDH fragment (internal control). RT-PCR products were resolved in 1.5% agarose gels stained with 1 μg/ml ethidium bromide. Gels were scanned by a Typhoon 8610 Scanner. Densitometric analysis was carried out with TotalLab software (Nonlinear Dynamics Ltd, Newcastle upon Tyne, U.K).

### Western blot

Lung tissue or isolated DLE cells from newborn rats in each experimental group was homogenized in T-Per™ Reagent (Pierce, Rockford, IL) containing protease inhibitors, aprotinin (30 μg/ml; Sigma, St. Louis, MO) and leupeptin (1 μg/ml; Sigma), with a homogenizer (Tissue Tearor) on ice. DLE cells were harvested in T-Per™ reagent and lysed by sonication. The homogenate was centrifuged at 13,000 *g *for 5 min at +4°C. Supernatant (membrane and cytosol) was collected, aliquoted in multiple vials, and snap-frozen in liquid nitrogen. One vial was used for determining total protein concentration of the sample to ensure equal loading of the electrophoresis gel. Aliquots were stored at -80°C until analyzed.

Polyacrylamide gel electrophoresis and transfer to nitrocellulose membranes (Pierce) were carried out using standard protocols. After the polyacrylamide gel electrophoresis and transfer, the nitrocellulose membranes were blocked (SuperBlock™ Dry Blend blocking buffer in tris buffered saline (TBS); Pierce) for 1 h at room temperature. After blocking, membranes were incubated with primary antibodies on an orbital shaker over night at +4°C. Primary αENaC antibodies were purchased from Alpha Diagnostics International (San Antonio, TX; used at 1:1,000 dilution) and directed against N-termini of αENaC. The antibodies recognize membrane proteins of appropriate sizes (85–95 kDa) in rats. Primary CFTR antibodies were bought from Santa Cruz Biotech, Inc. (Santa Cruz, CA; used at 1:1,000 dilution) and directed against amino acids 1–182 mapping the CFTR N-terminus. The antibody recognizes a membrane protein of appropriate size (~150 kDa) in rats. Monoclonal anti-GAPDH antibodies (GAPDH used as loading and transfer control; 1:1,000 dilution) were purchased from Cell Signaling Technology, Inc. (Danvers, MA), detects GAPDH of rat origin, and cross-reacts with guinea pig GAPDH (37 kDa). After incubation, membranes were washed 5 × 10 min with wash buffer (pH = 7.5; TBS with 0.1% Tween-20). Membranes were incubated with HRP-conjugated secondary antibodies (goat-anti-rabbit IgG; used at 1:1,000 dilution) for 1 h at room temperature. After incubation, membranes were washed again. Substrate solution (SuperSignal^® ^West Femto; Pierce) was added and incubated for 5 min. The luminescence signal was detected using a Kodak image analyzer and densitometrically analyzed using TotalLab software.

### Extravascular lung water

To measure extravascular lung water in newborn rat lungs, we modified the original method described previously [25]. Extravascular lung water was measured in untreated (*N *= 20), pSi-0-injected (*N *= 10), pSi-4-injected (*N *= 12), and pSi-C_2_-injected (*N *= 15) newborn rats from 3–4 litters each. The lungs were rapidly excised, hearts removed, and placed in pre-weighed sample tubes and re-weighed. Water (250 μl) was added, lungs were weighed again, and homogenized using a Tissue Tearor. If the extravascular lung water determinations were not done the day of lung harvest, collected lungs were weighed, water (250 μl) added, and lungs were re-weighed and stored frozen at -20°C until analysis. Parts of lung homogenates were centrifuged 5 min at 14,000 *g*. Blood was collected from a small number of newborn rats after decapitation to obtain a hemoglobin (Hb) value for newborn rat blood. Hb content was measured on supernatants obtained after centrifugation and blood volume of newborn rat lungs were calculated from homogenate supernatant Hb concentration relative to blood Hb concentration. Newborn rat blood wet-dry weight was determined. Lung wet-to-dry weights were corrected for blood volume. Drying of lung homogenates, lung homogenate supernatants, and newborn rat blood was carried out using a moisture analyzer (Sartorius, Edgewood, NY) that continuously recorded water loss as samples dried. Each sample was dried at 80–120°C until dry weights reached stability. Typically, this procedure required 15 min/sample. Non-specific water loss of wet samples and non-specific re-humidification of dried samples, as may occur when small samples are measured by traditional extravascular lung water techniques, was prevented in this analysis. We verified the technique by comparing it to traditional techniques [25] in adult rat lungs.

### Statistics

All data are presented as means ± SD. Data were analyzed with one-way analysis of variance (ANOVA) with Tukey's test as *post hoc *or Student's *t *test as appropriate. Differences were considered significant when *P *< 0.05.

## Results

### Lung CFTR mRNA during development

**Figure 1:**
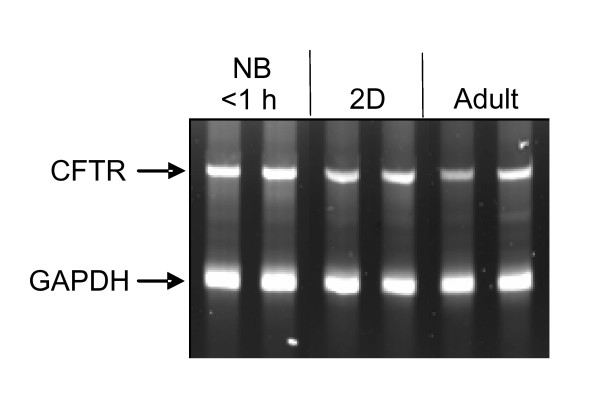
**CFTR mRNA during early postnatal life of the rat to adulthood.** CFTR mRNA was measured by RT-PCR in lung tissue from newborn (NB), 2-day-old (2D), and adult rats. Representative RT-PCR gels are shown for lung CFTR and GAPDH (internal standard) mRNA.

We determined if CFTR transcription changed during early postnatal development. Lung total RNA from newborn, 2-day-old (D), and adult rats were isolated. CFTR mRNA was determined by RT-PCR. GAPDH was used as internal control. CFTR transcription was ~2× higher in newborn than in adult rats (Fig. 1). CFTR transcription levels decreased during the first postnatal days and reached adult levels on postnatal day 2.

**Figure 2:**
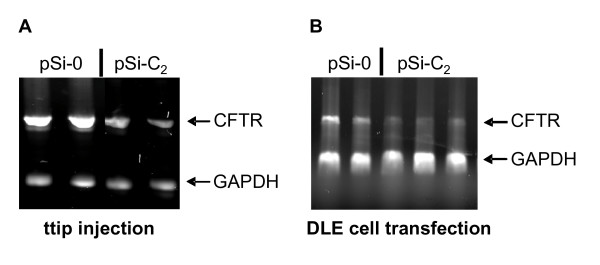
**CFTR mRNA in newborn rat lungs 24 h after ttip pSi-C_2_-injection (**A**) and CFTR mRNA in isolated DLE cells 24 h after pSi-C_2_-transfection (**B**).** Representative RT-PCR gels are shown for lung CFTR and GAPDH (internal standard) mRNA.

We then investigated if the selected siRNA-generating pDNA silenced CFTR both *in vivo *after ttip injection and *in vitro *in isolated DLE cells to assure its functionality for the further studies. We found that pSi-C_2 _was equally effective under either optimized condition to silence CFTR expression in both whole lung and DLE cells (Fig. 2AB).

### RNAi for CFTR in newborn rat lungs and isolated DLE cells

**Figure 3:**
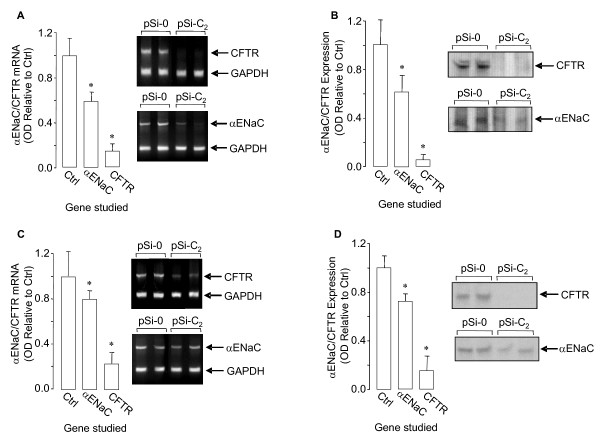
**CFTR and αENaC mRNA (A) and protein (B) 24 h after ttip pSi-C_2_-injection in lung homogenate from newborn rats.** Representative RT-PCR gels and western blots are shown for lung homogenate CFTR, αENaC, and GAPDH (internal standard) mRNA and protein. CFTR and αENaC mRNA (C) and protein (D) 24 h after transfection of isolated and cultured DLE cells with pSi-C_2_. Representative RT-PCR gels and western blots are shown for DLE cell CFTR, αENaC, and GAPDH (internal standard) mRNA and protein.

We also carried out a comprehensive study to determine if pSi-C_2_silenced CFTR in newborn rats. After ttip pSi-C_2_-injection, as shown in Fig. 3A, CFTR mRNA was decreased by ~80%. Western blot results demonstrated that pSi-C_2 _also decreased CFTR protein by ~80% (Fig. 3B). We then investigated if CFTR-silencing affected αENaC expression. As shown in Fig. 3A and Fig. 3B, CFTR-silencing also reduced αENaC mRNA and protein.

We turned our attention to confirming this in isolated DLE cells from rat fetuses. We therefore studied RNAi silencing of CFTR in these isolated DLE cells after pSi-C_2 _pretreatment. DLE cells (isolated at GD21) were transfected with the pDNA 1 day after cell plating. Twenty-four hours after pDNA transfection, i.e., GD22 (birth), CFTR and αENaC mRNA and protein were detected by RT-PCR and western blot, respectively. As shown in Fig. 3C, pSi-C_2_-transfection decreased CFTR mRNA by ~80%, compared to pSi-0 transfected matched DLE cells. As seen in Fig. 3D, western blot results emonstrate that, pSi-C_2_-transfection also decreased CFTR protein by ~90%.

### RNAi for αENaC in newborn rat lungs and isolated DLE cells

**Figure 4:**
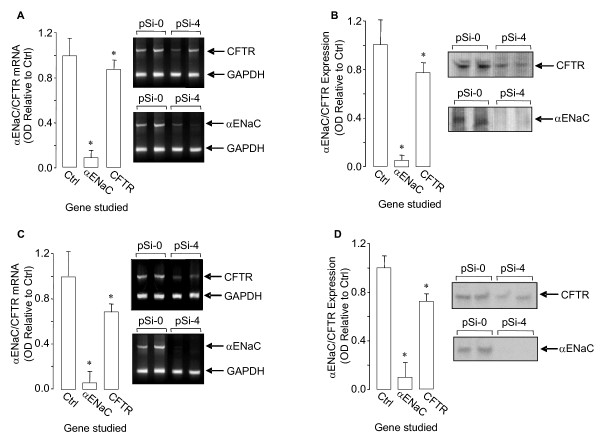
**αENaC and CFTR mRNA (A) and protein (B) 24 h after ttip pSi-4-injection in lung homogenate from newborn rats.** Representative RT-PCR gels and western blots are shown for lung homogenate αENaC, CFTR, and GAPDH (internal standard) mRNA and protein. αENaC and CFTR mRNA (C) and protein (D) 24 h after transfection of isolated and cultured DLE cells with pSi-4. Representative RT-PCR gels and western blots are shown for DLE cell αENaC, CFTR, and GAPDH (internal standard) mRNA and protein.

We determined if pSi-4 silenced αENaC in the newborn rats. After ttip pSi-4-injection, as shown in Fig. 4A, αENaC mRNA was decreased by ~90%. Western blot results demonstrated a decrease in αENaC protein by ~85% (Fig. 4B). We then investigated if αENaC-silencing affected CFTR expression; as shown in Figs. 4A and 4B, αENaC-silencing slightly reduced CFTR mRNA and protein.

We then studied RNAi silencing of αENaC in isolated DLE cells after pSi-4 pretreatment. DLE cells (isolated at GD21) were transfected as above with the pDNA 1 day after cell plating. Twenty-four hours after pDNA transfection, i.e., GD22 (birth), αENaC and CFTR mRNA and protein were detected by RT-PCR and western blot, respectively. As shown in Fig. 4C, pSi-4-transfection decreased αENaC mRNA by ~90%. As seen in Fig. 4D, western blot results demonstrate that, pSi-4-transfection also decreased αENaC protein by ~90%. Similar to the *in vivo *situation, CFTR mRNA and protein were less affected by the αENaC silencing.

### Extravascular lung water

**Figure 5:**
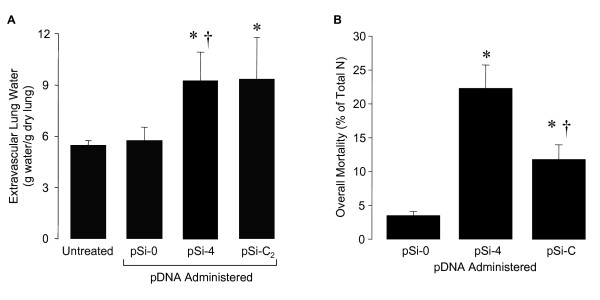
**Extravascular lung water (A) in newborn rats 24 h after ttip pSi-0- and pSi-C_2_-injections compared to untreated normal newborn age-matched rats.** Mortality (B) in newborn rats 24 h after ttip pSi-0- and pSi-C_2_-injections compared to untreated normal newborn age-matched rats.

Extravascular lung water was measured as a functional physiologic endpoint from ttip CFTR *in vivo *silencing. pSi-0-injected newborn rats displayed the same extravascular lung water as normal, untreated newborn rats. Extravascular lung water in ttip αENaC- and CFTR-silenced newborn rat lungs were both significantly increased after siRNA-generating pDNA injection (Fig. 5A).

### Mortality from in vivo CFTR-silencing

We then studied if ttip pSi-0-, pSi-4, or pSi-C_2_-injection affected newborn rat mortality. Newborn rats that died within 1 h after ttip injection were excluded as injection-related abnormalities (~3–4/litter irrespectively of experimental group). When tabulated, newborn rats that died after >1 h demonstrated the following mortality: pSi-0: 1 of 32 rats died, pSi-4: 9 of 40 rats died, and pSi-C_2_: 5 of 43 rats died. The data in Fig. 5B demonstrate mortality rates of <3% in ttip pSi-0-injected newborn rats, ~23% in ttip pSi-4-injected newborn rats, and ~12% in ttip pSi-C_2_-injected newborn rats.

### Localization of RNAi silencing

**Figure 6:**
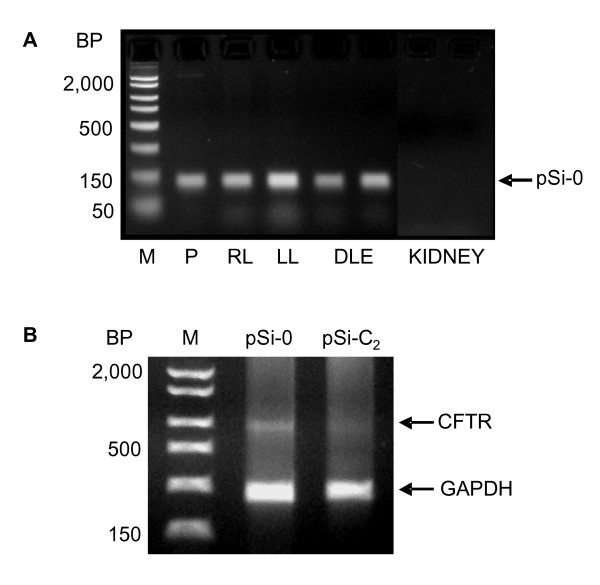
**Localization of pSi-0 pDNA 24 h following ttip pSi-0-injection in newborn rat lungs, in isolated DLE cells, and in the kidney (A) and identifying of the specific CFTR-silencing to the DLE cells 24 h after *in vivo *ttip administration (B).** A representative RT-PCR gel is shown for lung CFTR and GAPDH (internal standard) mRNA. (BP: base pairs; M: marker; P: purified plasmid; RL: right lung; LL: left lung)

To determine *in vivo *and *in vitro *transfection ability of pDNA during our conditions, we detected pSi-0 pDNA lung and kidney presence by PCR. As shown in Fig. 6A, there was a single clear band representing pSi-0 in both ttip pSi-0-transfected lung tissue (equal strength in both right and left lungs) and pSi-0-transfected DLE cells. The same band was completely absent from the kidney samples, thus indicating no expression of our siRNA-generating pDNA (pSi-0) in this organ.

We also examined alveolar distribution of siRNA-generating pDNA 24 h after ttip pDNA injection for CFTR. DLE cells were isolated from 6 pSi-0- and 6 pSi-C_2_-transfected newborn rats. As can be seen in Fig. 6B, CFTR mRNA, as determined by RT-PCR, was absent from isolated DLE cells after ttip pSi-C_2_-injection.

### Organ specificity of RNAi silencing

**Figure 7:**
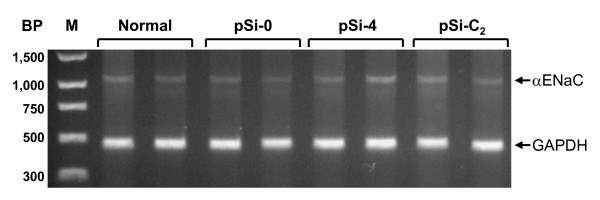
**αENaC mRNA in normal untreated and 24 h after ttip pSi-0-, pSi-4-, and pSi-C_2_-injection in kidney homogenate from newborn rats.** A representative RT-PCR gel is shown for kidney homogenate αENaC mRNA. (BP: base pairs; M: marker)

To determine organ specificity of the RNAi silencing of CFTR and αENaC *in vivo *in normal untreated newborn rats and in newborn rats 24 h after ttip pSi-0, pSi-4, and pSi-C_2_, we collected kidneys from these rats. In these kidneys, we determined if αENaC mRNA varied significantly between our groups. As shown in Fig. 7, there were similar expression of αENaC mRNA irrespectively which siRNA-generating pDNA that was used.

## Discussion

There were four important findings in our studies. First, ttip injection of specific CFTR siRNA-generating pDNA (pSi-C_2_) increased extravascular lung water and mortality rate of newborn rats. Second, ttip pSi-C_2_-injection decreased CFTR mRNA and protein in both *in vivo *newborn rat lungs and isolated, pSi-C_2_-transfected DLE cells. Third, CFTR-silencing by ttip pSi-C_2_-injection was specific. Fourth, ttip CFTR-silencing also reduced αENaC mRNA and protein expression, thus suggesting involvement of CFTR in regulation of ENaC at the conversion from lung fluid secretion to fluid absorption near term. Fifth, ttip αENaC silencing caused a slight reduction in CFTR mRNA and protein expression, further supporting a role for both proteins in the development of lung fluid absorption mechanisms.

The majority of infants make the transition from intrauterine life to postnatal life without complications, but only hours before birth, lungs are filled with an essentially protein-free isosmolar solution that has been actively secreted by the lung epithelium. The normal rate of lung fluid absorption in newborn rats has been determined earlier [12] and was not apparent before birth, reached a high rate immediately after birth, and decreased to the rate seen in adult rats by 40 h of newborn life. The molecular mechanism responsible for perinatal lung fluid absorption has been proposed to be ENaC, as mice deficient in αENaC expression dies within 40 h of birth from failure to clear fetal lung fluid [8]. mRNA for αENaC is found earliest at GD19, while both β and γ subunits are expressed at or after birth [26]. This expression patterns agrees well with the observed amiloride-sensitivity and function of fetal rat lungs at birth in the earlier study [12], especially since ENaC requires all three subunits to become fully functional. Recent studies have demonstrated that failure in lung fluid absorption at birth may be associated with ENaC deficiency [27-29]. In some cases, such as congenital diaphragmatic hernia (CDH), ENaC deficiency may have serious impact on lung fluid absorption at birth and drastically affect the ability to oxygenate the newborn [29]. In our current study, silencing CFTR with pSi-C_2 _was associated with elevated extravascular lung water and an increased mortality rate, thus strengthening the assumption of CFTR being involved in the transition from fluid-filled fetal lungs to air-filled newborn lungs at birth. ENaC and Cl transport proteins, such as CFTR, are two important membrane components expressed in the epithelial lining of lung alveoli [30]. An earlier rat study has reported that fetal lung fluid absorption was mediated by βAR stimulation [12]. However, the elevated lung fluid absorption rate in GD22 rat fetuses was only minimally amiloride-sensitive and increased in amiloride-sensitivity during the first 40 h of postnatal life [12]. In the current study, we investigated how CFTR transcription changed during early postnatal development. CFTR maintained a relatively high expression at birth and reached adult levels at the 2^nd ^postnatal day.

In our earlier study [9], αENaC gene silencing in adult rat lungs was achieved by siRNA generating pDNA, where the pDNA was delivered conjugated with liposomal complexes by intratracheal instillation. This method was originally developed by Folkesson and colleagues [31] and took advantage of the anatomical characteristics of the lung. To deliver pDNA in the original study [9] we utilized a modification of the discoveries by Sawa and colleagues [32], where they demonstrated that intraluminal water instillation into the lung increased transfection efficiency. However, this instillation technique is not suitable for newborn rats due to their small size. It has been demonstrated that DNA can be directly delivered to skeletal muscle by intramuscular injection of 'naked' pDNA [33]. The gene transfer was, however, restricted to muscle cells adjacent to the route of injection [33]. A more recent study by Bhargava and colleagues [34] demonstrated that site-specific transient gene knockdown can be achieved by local double-strand RNA hypothalamic injection. In our current study, we modified the siRNA-delivery methods by developing a repeatable ttip injection technique using a pDNA (μg):liposome (μl) ratio 1:1 with a pDNA concentration 4 μg/g body wt in a final volume of 40 μl/g body wt and with a low osmolality of 100 mOsm. Our results showed a reproducible specific siRNA-mediated αENaC and CFTR-silencing, about ~80–85% for both mRNA and protein, in newborn rat lungs by this method. In addition, our method was organ specific and did not affect ENaC expression in the kidney, another organ where ENaC is highly expressed, nor did the pDNA itself reach the kidneys. We also demonstrated that our siRNA-generating pDNAs did not reach the kidney after the ttip injections. In our recent publication [23] using this technique, we demonstrated the involvement of Nedd4-2 in ENaC membrane regulation and the importance for newborn lung conversion for fluid secretion to absorption in the rat.

An additional limitation of our data might be that the delivery of the siRNA-generating pDNAs invoked an interferon and/or cytokine response. Since both ENaC and CFTR can be affected by cytokines and interferons [35,36], this could potentially explain the downregulation of ENaC when the CFTR was silenced. However, for multiple reasons we do not believe this to be the case. First, since the interferon response is a response to the introduction of a siRNA to the cells, pSi-0 should also have caused a downregulation of ENaC and CFTR. This never occurred in these or our earlier studies [9,23]. Second, albeit more speculative, earlier studies suggest that introduction of pure siRNA directly to the cells were more likely to cause an interferon response than the introduction of siRNA-generating pDNA or phage-mediated transfection [37-39]. Third, all ENaC subunits, α-, β-, and γ, would likely have been downregulated when αENaC was silenced had there been an interferon response of significance. Fourth, in our earlier study [23], when Nedd4-2 was silenced, the expression of ENaC increased. Thus all this evidence argues against that a major interferon and/or cytokine response would be occurring in these studies.

As a functional endpoint, we evaluated changes in extravascular lung water and mortality following ttip pSi-C_2_. Interestingly, extravascular lung water was increased after ttip pSi-C_2_-injection. Moreover, pSi-C_2_-injection resulted in an increased mortality. These results indicate that CFTR is involved in the transition from lung fluid secretion to fluid absorption at birth. αENaC silencing resulted in a similar increase in extravascular lung water, with an even higher increase in mortality. The mortality rate, however, was not the same as αENaC gene knockout mice studies [8], possibly because the siRNA-generating pDNA was administered after birth, in contrast to a gestational knockout. It may also be associated with the fact that ttip pSi-4 and pSi-C_2_-injection only silenced αENaC and CFTR in the lung and thus we avoided unspecific systemic side-effects from αENaC and CFTR knockdown in other organs, i.e., the GI tract. A third possibility is that siRNA-mediated αENaC and/or CFTR knockdown was incomplete and left residual αENaC and CFTR expression in the lung. When a rescue model with CMV promoter-driven rat αENaC expression in the -/- αENaC mouse lung was utilized, a very low αENaC expression was encountered and this was apparently sufficient to rescue ~50% of the mouse pups at birth [40]. The mortality may also be attributable to that CFTR knockdown reduced αENaC mRNA and protein by ~20–40%, since ENaC deficiency is lethal.

Factors implied to regulate lung fluid absorption and ENaC expression in the newborn include βAR agonists, glucocorticoid hormones, thyroid hormones, and oxygen concentrations [12,41-44]. Key Na transport proteins involved are the basolateral Na,K-ATPase, that provides the driving force, and ENaC provides an apical pathway for Na entry into the epithelial cells [30]. Evidence has been brought forward suggesting that ENaC activation requires CFTR Cl channel function [45,46]. A number of hypotheses have been presented how CFTR control ENaC activity [47]. Research supports the hypotheses that there are ENaC-CFTR interactions related to electrical coupling of ion fluxes in epithelial cells [48]. Regulation of apical Na channels (ENaC and other Na channels) has been attributed to changes in cytosolic Ca, Cl concentration, and pH [49]. Our results indicate that ENaC and CFTR expression levels appear to correlate with each other at this critical stage of lung development. However, if one is affected by gene silencing (artificially or naturally by mutation), a functional deficiency of this channel may also affect the function/expression other channels. Potentially this may be explained as that ion flow through the channel, either partially or wholly, depends on the electrochemical potential. Thus, when two channels are active in the same membrane they would benefit from being able to influence each other's expression/function. The "electrical coupling" of Cl and Na conductances require that these two conductances are expressed in parallel at the apical epithelial cell membrane with a significant impact on each other. Our results also suggest an "expression coupling" that support and may provide some molecular background of the "electrical coupling" between ENaC and CFTR.

Was this "expression coupling" between ENaC and CFTR an "animal-only phenomenon" or could it be reproduced in simpler system such as cell systems? To answer this question we isolated and cultured DLE cells and transfected them *in vitro *with pSi-4 or pSi-C_2 _pDNA. DLE cell isolation was done on GD21 fetuses, transfection was done 1 day later, and αENaC and CFTR expression measured after another day of incubation. We decided on this protocol since it mimicked the time points when the newborn rats were transfected *in vivo*. Transfection of DLE cells with pSi-C_2 _resulted in a knockdown of CFTR, as observed in the newborn rat *in vivo*. Moreover, when pSi-C_2_-transfected cells were assayed for αENaC expression, we found that αENaC mRNA and protein were all decreased, also as observed in the newborn rats. Reversely, when the DLE cells were transfected with pSi-4, αENaC expression was decreased very significantly, while CFTR expression was much less affected. Thus, the "ENaC-CFTR expression coupling" was indeed present in the isolated DLE cells.

In conclusion, our data demonstrate by using selective siRNA inhibition of CFTR expression that both CFTR and ENaC are involved in the transition from lung fluid secretion to lung fluid absorption at birth. Our data also suggest that ENaC, being the principal agent stimulating lung fluid absorption at birth, may possibly depend on CFTR expression/function. Both CFTR and ENaC seem to be interdependent to each other in order to generate the driving force for perinatal lung fluid absorption.

## Competing interests

The authors declare that they have no competing interests.

## Authors' contributions

TL and SK carried out the experimental studies and drafted the manuscript. HGF designed the experimental setup, supervised the work, and provided intellectual input for the manuscript preparation. All authors have read the final version and approved it.
